# The Pathophysiological Role of Mitochondria-associated Membranes in Coronary Artery Disease and Atherosclerosis

**DOI:** 10.2174/0109298673343245250128093845

**Published:** 2025-02-12

**Authors:** Junyan Zhang, Zhongxiu Chen, Li Rao, Yong He

**Affiliations:** 1 Department of Cardiology, West China Hospital of Sichuan University, Chengdu, China

**Keywords:** Mitochondria-associated membranes (MAMs), coronary artery disease (CAD), atherosclerosis, reactive oxygen species (ROS), ischemia-reperfusion injury, autophagy, calcium homeostasis

## Abstract

Mitochondria-associated membranes (MAMs) are pivotal in cellular homeostasis, mediating communication between the endoplasmic reticulum and mitochondria. They are increasingly recognized for their role in atherosclerosis and coronary artery disease (CAD). This review delves into the cellular perspective of MAMs' impact on atherosclerosis and CAD, highlighting their influence on disease progression and the potential for therapeutic intervention. MAMs are implicated in key pathophysiological processes such as the generation of reactive oxygen species, calcium homeostasis, myocardial ischemia-reperfusion injury, autophagy, lipid synthesis and transport, and energy metabolism—fundamental to the development and progression of atherosclerosis and CAD. The complex interplay of MAMs with these pathological processes underscores their potential as therapeutic targets. This review synthesizes current understanding and emphasizes the need for further research to elucidate the multifaceted roles of MAMs in atherosclerosis and CAD, offering avenues for developing novel strategies aimed at improving mitochondrial health and mitigating the impact of these conditions.

## 1 INTRODUCTION

Mitochondria-associated membranes (MAMs) are specialized regions of the cell where the endoplasmic reticulum (ER) and mitochondria interact, serving as critical hubs for cellular functions such as calcium signaling, the regulation of reactive oxygen species (ROS) and lipid metabolism, *etc.* [1, 2]. Their strategic positioning and multifunctional roles underscore their importance in maintaining cellular homeostasis and their potential as central regulators of various physiological and pathological processes [3, 4].

Atherosclerosis and coronary artery disease (CAD) are leading causes of global morbidity and mortality, characterized by complex pathophysiological mechanisms that involve both metabolic dysregulation and inflammatory responses [5-7]. The interplay between MAMs and these pathological processes has begun to be elucidated, with evidence suggesting that MAMsplay a significant role in the development and progression of atherosclerosis and CAD [2, 3, 8]. Their involvement in key pathophysiological processes, such as ROS production [9, 10], calcium overload [1, 11, 12], and autophagy [13, 14], *etc.*, positions MAMs as potential therapeutic targets of CAD. This review aims to provide the current understanding of MAMs' impact on atherosclerosis and CAD, highlighting their role in disease progression and the potential for therapeutic intervention.

## 2 STRUCTURAL AND FUNCTIONAL OVERVIEW OF MAMS

**Fig. (1):**
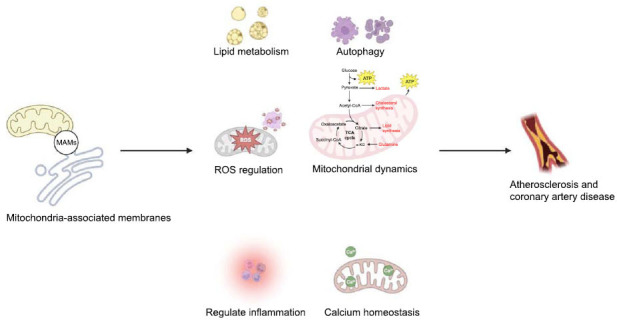
The role of MAMs in atherosclerosis and coronary artery disease.

The ER and mitochondria are interconnected organelles that play crucial roles in cellular homeostasis. In the absence of direct membrane fusion, the ER and mitochondria uphold a stable yet dynamic communication facilitated by proteins serving as tethers linking these two organelles. MAMs are specialized microdomains where the ER and mitochondria establish close physical and functional connections. These MAMs play significant roles in CAD and atherosclerosis and serve as crucial hubs that integrate diverse cellular processes, including lipid metabolism, calcium homeostasis, autophagy, mitochondrial dynamics, inflammation, Li *et al.* (Fig. **1**) [3].

The constitution of MAMs demonstrates notable dynamism, incorporating in excess of 1000 proteins actively involved in these intricate cellular mechanisms [4]. We summarize the most relevant molecules and functions associated with MAMs in relation to atherosclerosis and CAD, highlighting their potential as therapeutic targets (Table **1**).

## 3 MAMS AND ROS PRODUCTION IN CAD

**Fig. (2):**
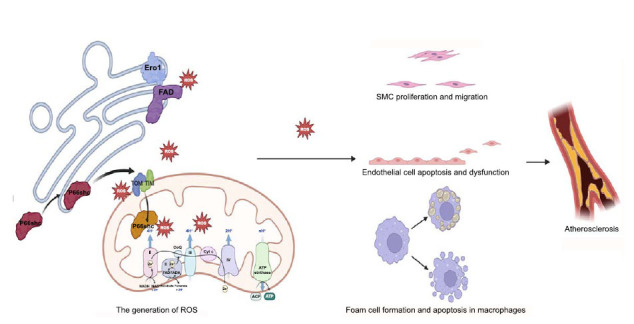
The role of MAM in ROS production and its impact on atherosclerosis and CAD.

Atherosclerosis is delineated as a pathophysiological process entailing lipid accumulation and immune responses, with oxidative stress provoked by ROS playing a pivotal role in disrupting oxidative equilibrium, thereby emerging as a crucial element in atherosclerosis pathophysiology (Fig. **2**) [15]. ROS inflict damage on the vascular wall and modulate the formation and stability of atherosclerotic plaques through their involvement in or regulation of critical cellular functions and biological pathways, including the oxidative modification of lipoproteins, the proliferation and migration of smooth muscle cells, cellular inflammatory responses, and necrosis [16]. Consequently, strategies aimed at mitigating ROS production or augmenting antioxidant defense mechanisms represent promising therapeutic avenues in the management of atherosclerosis [17]. The involvement of ROS in the key processes of atherosclerosis across different cell types is presented in Table **2**.

The relationship between MAMs and ROS production can be divided into two aspects. Firstly, certain molecules within the MAMs structure, such as Ero1 and p66Shc, directly contribute to ROS production. Ero1, including Ero1-α and Ero1-β, with 75% of Ero1-α localized on MAMs, plays a crucial role in controlling the redox state of the endoplasmic reticulum, and increased expression leads to elevated ROS production [18]. p66Shc is primarily found in the mitochondrial matrix, between the inner and outer mitochondrial membranes, and on the outer mitochondrial membrane. Dephosphorylation of the Ser36 site on p66Shc enables its translocation to MAMs, where it mediates ROS production [10]. On the other hand, ROS promotes the flow of calcium ions from the ER to mitochondria through MAMs [19]. The accumulation of mitochondrial calcium ions leads to mitochondrial depolarization and abnormal oxidative phosphorylation, resulting in the uncoupling of the electron transport chain and respiratory complexes I and III, further increasing mitochondrial ROS production [20, 21]. Inhibitors of ER calcium ion channels can block this process [9].

Oxidative stress, driven by uncontrolled ROS production, propagates inflammation, endothelial dysfunction, and the progression of atherosclerotic lesions, which are hallmark features of CAD [22, 23]. The dual role of voltage-dependent anion channel 1(VDAC1), as both a mediator of cellular metabolism and a contributor to ROS-induced cardiac damage, situates this protein as a focal point in the study of cardioprotective strategies. Inhibition of VDAC1 expression or its phosphorylation states offers a potential avenue for reducing ROS levels and preventing mitochondrial and cardiac dysfunction [24].

In summary, MAMs play a crucial role in the production of ROS, contributing to the development of atherosclerosis and CAD. Targeting MAMs and adjusting ROS levels could potentially serve as effective therapeutic strategies for managing CAD.

## 4 MAMS IN CALCIUM HOMEOSTASIS AND MYOCARDIAL ISCHEMIA-REPERFUSION INJURY

**Fig. (3):**
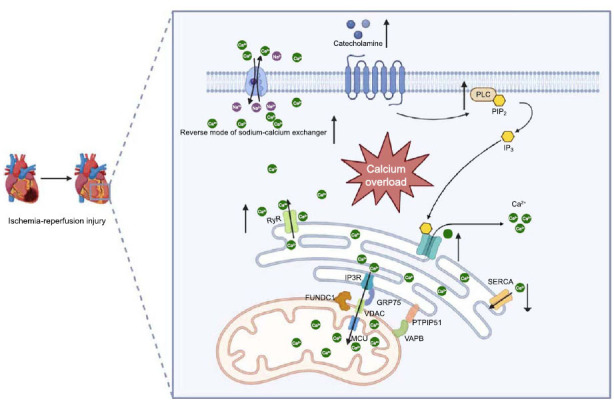
Molecules in MAMs are involved in calcium overload and myocardial ischemia-reperfusion injury.

One of the major unresolved issues in patients with coronary artery disease is myocardial ischemia-reperfusion injury following myocardial infarction and subsequent revascularization [25, 26]. This condition can lead to myocardial cell dysfunction or even cell death. Numerous studies have demonstrated that intracellular calcium overload is a key factor in the development of ischemia-reperfusion injury in myocardial cells [27-30]. MAMs play a crucial role in the regulation of intracellular calcium homeostasis and are indispensable in the context of ischemia-reperfusion injury (Fig. **3**, Table **3**).

Ischemia impairs the function of sarcoplasmic/ER Ca^2+^-ATPases (SERCAs), preventing these enzymes from re-sequestering calcium into the endoplasmic reticulum, thereby leading to cytosolic calcium accumulation [31, 32]. Concurrently, the reverse mode activity of the cell surface Na^+^/Ca^2+^ exchanger (NCX) is enhanced, further increasing cytosolic Ca^2+^ concentration and resulting in calcium overload [33, 34]. Additionally, during ischemia-reperfusion injury, the release of substances such as catecholamines increases, activating PLC-mediated signaling pathways, where inositol 1,4,5-trisphosphate (IP3), a downstream product, binds to IP3 receptors on the endoplasmic reticulum, opening calcium channels and raising intracellular calcium levels [35, 36]. Other MAM components, such as IP3Rs, VDACs, and GRP75, facilitate direct calcium transfer from the endoplasmic reticulum to mitochondria [1, 11, 12]. This proximity transfer enhances calcium ion transport into the mitochondria, leading to mitochondrial calcium overload.

Numerous studies have investigated the changes in MAMs components and their roles in calcium homeostasis during I/R. Qiao *et al.* showed that PTPIP51 levels increase in the hearts of mice after I/R, and knocking down PTPIP51 can improve cardiomyocyte function and reduce infarct size [37]. The ER protein VAPB interacts with PTPIP51, and downregulation of either protein elicits lower mitochondrial calcium levels and autophagy, protecting against I/R injury [38, 39]. Chemical chaperones like tauroursodeoxycholic acid and 4-phenylbutyrate have been found to improve related conditions by alleviating ER-mitochondria connection disruptions and ER stress [40]. Additionally, regulating mitochondrial calcium uniporter (MCU) and its regulatory proteins may influence mitochondrial calcium uptake and mitigate calcium overload during I/R [41].

Indeed, in addition to their role in calcium homeostasis, the various molecular components within the MAMs regulate numerous other pathways that modulate the response to I/R injury. For instance, MAMs can influence the generation of mitochondrial ROS, as well as mitochondrial fusion, fission, and autophagy processes [42, 43]. Future research should further elucidate the specific roles of MAM components in regulating various cellular processes during I/R injury, explore novel therapeutic targets within MAMs, and develop targeted interventions to restore cellular homeostasis and protect cells from I/R-induced death.

## 5 MAMS AND AUTOPHAGY IN CAD

Autophagy maintains cellular homeostasis by delivering damaged organelles and excess biomacromolecules to the lysosome for degradation, utilizing the degradation products for energy and cellular reconstruction [44]. MAMs play a crucial role in this process. Studies have shown that during starvation-induced autophagy, autophagic precursors form at the ER and outer mitochondrial membrane, with MAMs being essential for autophagosome formation [45]. Key proteins such as MFN2, ATG14, and ATG5 are localized at MAMs and are necessary for autophagy [46, 47]. The mechanistic target of rapamycin complex 2 (mTORC2), located at MAMs, regulates mitochondrial function and autophagy through PACS-2 and hexokinase phosphorylation, as well as IP3R3-mediated calcium release [48, 49]. Proteins like Rab32 and PINK1 at MAMs are also vital, with PTEN-induced kinase 1 (PINK1) promoting the contact between mitochondria and the ER, facilitating autophagosome precursor formation [50, 51]. Overexpression of protein kinase C β (PKCβ) and p66Shc reduces autophagic activity [49, 52]. These findings underscore the pivotal role of MAMs and associated proteins in the regulation of autophagy.

Autophagy is found in all cell types involved in atherosclerotic plaque formation (macrophages, VSMC, and endothelial cells), triggering inflammasome activation and ultimately leading to plaque accumulation (Table **4**). Although autophagy dysfunction is a common factor accelerating plaque progression, the underlying mechanisms differ across the cell types involved. For instance, aberrant autophagy in macrophages promotes plaque instability by increasing cell apoptosis and necrosis [14]; conversely, autophagy defects in VSMCs accelerate plaque formation through the induction of senescence [13]; endothelial cells lacking essential autophagy-related genes exhibit characteristics of both apoptosis and senescence [53]. These findings underscore the cell type-dependent mechanisms of autophagy in the pathogenesis and progression of atherosclerosis, highlighting the importance of developing autophagy-based therapeutic strategies for atherosclerosis in a cell-specific context.

Melatonin can reduce inflammation and play a positive role in stabilizing atherosclerotic plaques by activating the SIRT3/FOXO3a/Parkin-dependent mitochondrial autophagy pathway [54]. Reversing autophagy dysfunction in plaque macrophages through trehalose or overexpression of the transcription factor EB reactivates autophagy, diminishing apoptosis and pro-inflammatory signaling in plaque macrophages, effectively alleviating atherosclerotic lesions [55]. Similarly, *in vitro* experiments have shown that activating mitochondrial autophagy through overexpression of PINK1 or Parkin can protect vascular smooth muscle cells exposed to atherosclerosis-promoting conditions, thereby preserving cardiovascular health and disease [56]. These findings collectively suggest that targeting autophagy or mitochondrial autophagy activation could reduce the burden of atherosclerotic plaques, thereby offering protection against atherosclerotic cardiovascular diseases.

Multiple autophagy inducers, including trehalose and spermidine, have been shown to limit myocardial damage and improve the prognosis of ischemic heart injuries [57, 58]. Conversely, inhibiting autophagy pharmacologically (*e.g.*, with 3-methyladenine or bafilomycin A1) or genetically (*e.g.*, through mTOR activation or AMPK inhibition) exacerbates myocardial damage in mice with myocardial infarction and promotes adverse ischemic cardiac remodeling [59-61]. Furthermore, in mouse models, experimental groups with Parkin gene knockout, which reduces mitochondrial autophagy, are more sensitive to ischemic heart damage and exhibit larger infarct sizes and lower survival rates compared to control groups [62-64]. During reperfusion, despite the supplementation of oxygen and nutrients to the ischemic myocardium, excessive accumulation of ROS occurs [65, 66]. This overproduction of oxidative stress leads to the excessive activation of autophagy during reperfusion, thereby inducing myocardial cell death and exacerbating reperfusion injury [67, 68].

In summary, MAMs and autophagy play important roles in the pathophysiological processes of atherosclerosis and coronary artery disease. Developing therapeutic strategies that precisely regulate autophagy in different cell types could offer novel approaches for atherosclerosis prevention and treatment.

## 6 MAMS AND LIPID SYNTHESIS AND TRANSPORT IN CAD

**Fig. (4):**
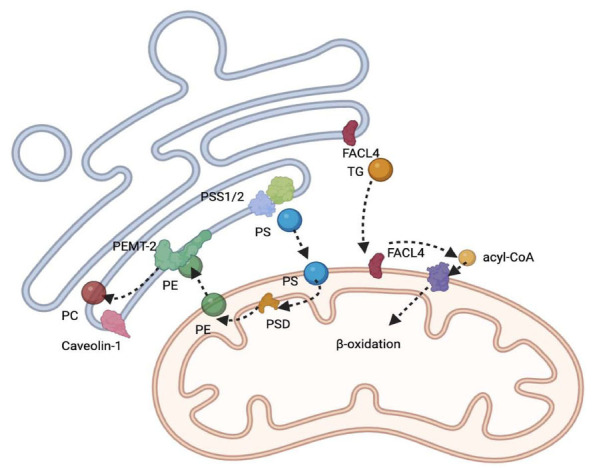
The role of MAMs in lipid metabolism.

MAMs have emerged as critical sites for lipid synthesis and transport, playing a pivotal role in the pathogenesis of atherosclerosis and CAD (Fig. **4**, Table **5**). The structural components of MAMs, including phosphatidylserine synthase 1 and 2 (PSS1/2), are essential for the biosynthesis of phosphatidylserine (PS) and its subsequent conversion to phosphatidylcholine (PC), a process integral to membrane dynamics and lipid homeostasis [69]. The translocation of PS through MAMs into mitochondria represents a rate-limiting step in the synthesis of phosphatidylethanolamine (PE), highlighting the significance of MAMs in phospholipid metabolism. Moreover, the presence of acyl-CoA synthetase long-chain family member 4 (ACSL4) and FACL4 within MAMs underscores their involvement in the synthesis of triglycerides and the regulation of steroidogenesis, respectively [70]. Notably, caveolin-1, identified in the MAMs of murine liver, plays a vital role in the metabolism of sterols and lipoproteins, further illustrating the multifaceted functions of MAMs in lipid regulation.

MAMs facilitate the transfer of lipids between the endoplasmic reticulum and mitochondria, with proteins such as PEMT2, PSS1/2, and FACL4 being crucial for the metabolism of phospholipids [71, 72]. This inter-organelle lipid transfer is critical for the synthesis of mitochondrial cardiolipin, a phospholipid essential for mitochondrial function and cardioprotection [73]. Additionally, MAMs are involved in the metabolic processing of cholesterol and ceramides, with the transfer of cholesterol to mitochondria being a key step in steroidogenesis [74]. The presence of specific components such as Caveolin-1(CAV1 in MAMs is instrumental in the intracellular transport of cholesterol, highlighting the complex interplay between MAMs and lipid homeostasis [75].

In conclusion, MAMs play a crucial role in the synthesis and transport of lipids, hosting multiple key enzymes involved in phospholipid biosynthesis [76]. Their involvement in the regulation of cholesterol and phospholipid metabolism has profound implications for the development of atherosclerosis, obesity, and insulin resistance, highlighting the potential of targeting MAMs function in the prevention and treatment of atherosclerosis and coronary heart disease [2, 77, 78].

## 7 MAMS AND CARDIAC ENERGY METABOLISM IN CAD

Under ischemic conditions, myocardial cells exhibit adaptive metabolic changes [79]. Mild ischemia primarily sees the heart utilizing the oxidative function of myocardial fatty acids; moderate ischemia accelerates anaerobic glycolysis and enhances FFA oxidation. As ischemia worsens, both FFA and glucose metabolism are inhibited, leading to increased lactate accumulation due to incomplete aerobic oxidation of pyruvate, causing intracellular acidosis [80]. Concurrently, the aerobic oxidation of fatty acids significantly declines or ceases under ischemic conditions, resulting in cellular and mitochondrial membrane damage, potentially leading to myocardial cell damage or death. Additionally, this condition suppresses the activity of pyruvate dehydrogenase, inhibiting glucose's aerobic oxidation and reducing ATP production efficiency [81]. This suppression contributes to decreased myocardial contractile function. Given that fatty acid metabolism produces less ATP than glucose pathways, regulating energy metabolism in ischemic myocardial tissue involves suppressing fatty acid oxidation and enhancing glucose oxidation [82].

MAMs serve as critical microdomains within the cellular architecture, pivotal for maintaining cellular integrity and energy metabolism. One of the key elements is pyruvate dehydrogenase kinase 4 (PDK4), a mitochondrial matrix enzyme that regulates cellular energy metabolism. In the calcified vascular smooth muscle cells of patients with atherosclerosis, an upregulation of PDK4 has been observed [83]. This upregulation compromises the integrity of MAMs, impairing mitochondrial respiratory function and subsequently inhibiting autophagy. Furthermore, PDK4 drives metabolic reprogramming, increasing the rate of glycolysis in vascular smooth muscle cells under calcifying conditions. Inhibition of glycolysis, conversely, promotes apoptosis of these cells [83].

Also, research has highlighted the importance of mitochondrial energy metabolism in ameliorating the pathological morphology of the myocardium [84]. Enhancing ATP content and regulating the opening of the mitochondrial permeability transition pore (mPTP) can modulate cardiac energy expenditure, inhibit apoptosis, and improve symptoms of myocardial ischemia [85]. The body adapts to coronary heart disease and myocardial ischemia by reducing cellular oxygen consumption and producing small amounts of ATP through glycolysis [86]. However, the ATP generated *via* glycolysis is insufficient to support normal physiological functions of the heart, leading to an accumulation of ROS during mitochondrial energy metabolism [87]. This accumulation further damages the mitochondria, disrupting the dynamic balance between oxidation and reduction, leading to abnormal opening of mPTP [88]. This disruption results in an aberrant distribution of charged ions across the membrane, loss of the electrochemical gradient, reduction of mitochondrial membrane potential, and the release of pro-apoptotic factors such as cytochrome c and Bax into the cytosol, thereby promoting myocardial cell apoptosis [89, 90].

In summary, MAMs are crucial in cardiac energy metabolism through their impact on mitochondrial function, autophagy, and apoptosis, necessitating further research into their molecular mechanisms and interactions with mitochondrial metabolism to develop novel strategies against atherosclerosis and CAD.

## 8 MAMS IN DRUG DELIVERY TO TREAT CAD

Recent advancements in nanotechnology have provided a variety of innovative approaches for mitochondrial-targeted drug delivery [91]. Common strategies include the use of charged nanoparticles, mitochondrial penetrating peptides (MTPs), and carriers modified with specific ligands, ensuring precise localization and translocation across the mitochondrial double membrane [92]. For instance, liposomes and metal nanoparticles have been extensively studied for delivering antioxidants and energy metabolism regulators, demonstrating the potential to alleviate oxidative stress and improve mitochondrial function [93, 94]. Additionally, recent research has explored the combination of nanodelivery systems with gene therapy, further enhancing therapeutic outcomes by regulating the expression of mitochondrial-related genes [95].

Despite the promising prospects of mitochondrial membrane-targeted drug delivery in the treatment of coronary artery disease, several challenges remain, such as the biocompatibility of carriers, delivery efficiency, and long-term safety concerns that need to be addressed. Future research should focus on developing more efficient and specific delivery carriers and validating their safety and efficacy through clinical trials. Furthermore, a deeper understanding of the particular pathological mechanisms of mitochondria in coronary artery disease will aid in designing more precise drug delivery strategies, thereby facilitating the translation and promotion of mitochondrial-targeted therapies in clinical applications.

## CONCLUSION

The review underscores the critical role of MAMs in the pathophysiology of atherosclerosis and CAD. MAMs are implicated in key pathophysiological processes such as the generation of ROS, calcium homeostasis, myocardial ischemia-reperfusion injury, autophagy, lipid synthesis and transport, and energy metabolism—fundamental to the development and progression of atherosclerosis and CAD. Targeting MAMs offers a promising therapeutic approach for the management of atherosclerosis and CAD. Future research should focus on dissecting the complex mechanisms by which MAMs interact with the pathophysiological processes in atherosclerosis and CAD, with the aim of identifying specific targets for intervention.

## Figures and Tables

**Table 1:** The main molecules involved in atherosclerosis and coronary artery disease within MAMs and their primary pathophysiological processes.

**Molecular**	**Main Pathophysiologic Processes in Atherosclerosis and Coronary Heart Disease**	**References**
IP3Rs	Calcium homeostasis, ischemia-reperfusion injury	[1, 11, 12]
GRP75	Calcium homeostasis, ischemia-reperfusion injury	[1, 11, 12]
VDACs	Calcium homeostasis, ischemia-reperfusion injury, autophagy	[1, 11, 12]
p66Shc	ROS production, autophagy	[10]
Ero1	ROS production	[18]
SERCAs	Calcium homeostasis, ischemia-reperfusion injury	[31, 32]
NCX	Calcium homeostasis, ischemia-reperfusion injury	[33, 34]
PTPIP51	Ischemia-reperfusion injury, autophagy	[38, 39]
VAPB	Ischemia-reperfusion injury, autophagy	[38, 39]
MFN2	Autophagy, mitochondrial dynamics	[47]
ATG14	Autophagy	[46]
ATG5	Autophagy	[46]
mTORC2	Autophagy, energy metabolism	[49]
PINK1	Autophagy, mitochondrial dynamics	[56]
BECN1	Autophagy	[50, 51]
Rab32	Autophagy	[48]
PKCβ	Autophagy	[49, 52]
Parkin	Autophagy	[56]
SREBP2	Lipid synthesis and transport	[96]
CAV1	Lipid synthesis and transport	[75]
ACSL4	Lipid synthesis and transport	[71, 72]
PEMT2	Lipid synthesis and transport	[71, 72]

**Abbreviations: **IP3Rs, Inositol 1,4,5-trisphosphate receptors; GRP75, Glucose-regulated protein 75; VDACs, Voltage-dependent anion channels; p66Shc, p66 isoform of the growth factor adapter Shc; Ero1, Endoplasmic reticulum oxidoreductin-1; SERCAs, Sarco/endoplasmic reticulum Ca^2+^-ATPases; NCX, Sodium-calcium exchanger; IP3, Inositol 1,4,5-trisphosphate; PTPIP51, Protein tyrosine phosphatase interacting protein 51; VAPB, VAMP (vesicle-associated membrane protein)-associated protein B; MFN2, Mitofusin 2; ATG14, Autophagy-related protein 14; ATG5, Autophagy-related protein 5; mTORC2, Mechanistic target of rapamycin complex 2; PINK1, PTEN-induced putative kinase 1; BECN1, Beclin-1; Rab32, Ras-related protein Rab-32; PKCβ, Protein kinase C beta; Parkin, E3 ubiquitin-protein ligase parkin; SREBP2, Sterol regulatory element-binding protein 2; CAV1, Caveolin 1; ACSL4, Long-chain-fatty-acid-CoA ligase 4; FACL4, Fatty acyl-CoA ligase 4; PEMT2, Phosphatidylethanolamine N-methyltransferase 2.

**Table 2:** The involvement of reactive oxygen species (ROS) in the key processes of atherosclerosis across different cell types.

**Cell Types**	**Role in Atherosclerosis**	**Role of ROS**	**Impact and Consequences**	**Potential Intervention Strategies**	**Reference**
Vascular smooth muscle cells	Abnormal proliferation and migration of vascular smooth muscle cells are involved in the formation of atherosclerosis.	Promote cell proliferation and migration, especially after cell damage	Exacerbating the progression of atherosclerosis	● Antioxidants (*e.g.*, chicoric acid, resveratrol) can reduce ROS production, inhibit the ROS/NFκB/mTOR/P70S6K signaling pathway, and slow cell proliferation and migration.● Hibiscus leaf polyphenols can inhibit the production of ROS, downregulate MMP-9, and relieve atherosclerotic lesions.	[97, 98]
Endothelial cell	Endothelial cells are responsible for maintaining and regulating the structure and function of blood vessels and play an important role in the early development and formation of atherosclerosis	Normally associated with NO levels, oxidative stress can lead to decreased NO bioavailability and increased ROS production, causing endothelial cell dysfunction.	Dysfunction may contribute to the early onset of atherosclerosis, including intercellular adhesion and aggregation in the vessel wall.	The use of antioxidants such as astragaloside reduces ROS that induces oxidative stress, prevents or reverses the uncoupling of eNOS, increases eNOS and NO as well as activates the antioxidant system.	[99-101]
Macrophage	Under ox-LDL conditions, macrophages take up the aggregated and oxidized LDL, leading to their transformation into foam cells and release inflammatory factors that promote the accumulation of monocytes, ultimately resulting in the formation of fibrous plaques with accumulated lipids and smooth muscle cells.	Highly expressed (Immunity-related GTPase family M protein) IRGM is associated with ROS and MAPK signaling pathways in macrophages from patients with atherosclerosis, and ROS-induced lipid peroxidation has a role in apoptosis and autophagy	IRGM expression is associated with necrotic formation of plaque cores, macrophage apoptosis, and autophagy. Excessive accumulation of ROS can also cause autophagic cell death.	Arsenic trioxide protects against atherosclerosis by inducing macro- phage autophagy through modulation of ROS-dependent TFEB nuclear translocation and the AKT/mTOR pathway.	[102, 103]

**Abbreviations: ** VSMC, vascular smooth muscle cells; ROS, reactive oxygen species;NFκB, nuclear factor kappa B; mTOR, mechanistic target of rapamycin; P70S6K, ribosomal protein S6 kinase beta; MMP-9, matrix metalloproteinase 9; eNOS, endothelial nitric oxide synthase; NO, nitric oxide; IRGM, immunity-related GTPase family M protein; MAPK, mitogen-activated protein kinase; TFEB, transcription factor EB; AKT, protein kinase B.

**Table 3:** Main molecules involved in ischemia-reperfusion injury within MAM and potential therapeutic strategies.

**Molecule/Component**	**Mechanism**	**Research Findings**	**References**
IP3Rs	Tethering protein facilitates direct transmission of calcium from the ER to mitochondria, increasing calcium transfer to mitochondria and contributing to calcium overload.	Dysregulation of IP3Rs plays a role in calcium transfer, further contributing to mitochondrial calcium overload.	[104]
VDACs	Tethering protein facilitates direct transmission of calcium from the ER to mitochondria, increasing calcium transfer to mitochondria and contributing to calcium overload.	Dysregulation of VDACs plays a role in calcium transfer, further contributing to mitochondrial calcium overload.	[105]
GRP75	Tethering protein facilitates direct transmission of calcium from the ER to mitochondria, increasing calcium transfer to mitochondria and contributing to calcium overload.	Dysregulation of GRP75 plays a role in calcium transfer, further contributing to mitochondrial calcium overload.	[106]
MCU	Responsible for the uptake of calcium into the mitochondrial matrix. Disturbed ER-mitochondria connection and ER stress affect calcium transfer efficiency *via* MCU.	Modulation of MCU and its regulatory proteins (*e.g.*, MiCU family members and MCUR1) can impact mitochondrial calcium uptake and alleviate calcium overload.	[41]
PTPIP51	Increases proximity between ER and mitochondria, promoting calcium transfer.	Elevated levels of PTPIP51 were observed in mouse hearts following ischemia-reperfusion challenge. Knockout of PTPIP51 ameliorates cardiomyocyte dysfunction and reduces infarct size.	[37]
VAPB	ER protein that interacts with PTPIP51 to regulate ER-mitochondria connection.	Downregulation of VAPB induces autophagy and lowers mitochondrial calcium levels, protecting against I/R injury.	[39]

**Abbreviations: **IP3Rs, Inositol trisphosphate receptors; VDACs, Voltage-dependent anion channels; GRP75, Glucose-regulated protein 75; MCU, Mitochondrial calcium uniporter; MiCU, Mitochondrial calcium uniporter family members; MCUR1, Mitochondrial calcium uniporter regulatory protein 1; PTPIP51, Protein tyrosine phosphatase interacting protein 51; VAPB, Vesicle-associated membrane protein-associated protein B.

**Table 4:** The role of Autophagy Dysfunction in different cells in atherosclerosis.

**Cell Type**	**Role of Autophagy Dysfunction**	**Consequences**	**References**
Endothelial Cells	Endothelial cells lacking essential autophagy-related genes exhibit both apoptosis and senescence, leading to impaired endothelial barrier function, increased inflammation, and the promotion of atherosclerosis.	Promote atherosclerosis	[53]
Smooth Muscle Cells	Autophagy-deficient vascular smooth muscle cells promote plaque formation through senescence induction.	Promote atherosclerosis	[13]
Macrophages	Abnormal autophagy in macrophages promotes plaque instability by increasing apoptosis and necrosis, thereby exacerbating the progression of atherosclerosis.	Promote atherosclerosis	[14]

**Table 5:** Key enzymes and related pathways of different types of lipids in MAMs.

**Lipid Type**	**Key Enzyme in MAMs**	**Main Pathways Involved**	**References**
PS	PSS1/2	CDP-choline pathway and PS decarboxylation pathway	[71, 72]
PE	PSS1/2	Similar to PS synthesis	[71, 72]
TG	DGAT	Glycerol monoacyltransferase and glycerol di acyltransferase pathways	[107]
Cholesterol	CAV1	Cholesterol transport	[75]

**Abbreviations: **PS, Phosphatidylserine; PE, Phosphatidylethanolamine; TG, Triacylglycerol;FACL4, Fatty acyl-CoA ligase 4;CAV1, Caveolin 1;PSS1/2, Phosphatidylserine synthase 1/2;DGAT, Diacylglycerol acyltransferase.

## LIST OF ABBREVIATIONS

MAMs: Mitochondria-associated Membranes
ER: Endoplasmic Reticulum
CAD: Coronary Artery Disease
ROS: Reactive Oxygen Species
SERCAs: Sarcoplasmic/Endoplasmic Reticulum Ca^2+^-ATPases
NCX: Na^+^/Ca^2+^ Exchanger
PLC: Phospholipase C
IP3: Inositol 1,4,5-trisphosphate
IP3Rs: Inositol 1,4,5-trisphosphate Receptors
VDAC1: Voltage-dependent Anion Channel 1
GRP75: Glucose-regulated Protein 75
PTPIP51: Phosphatase and Tensin Homolog-interacting Protein 51
VAPB: Vesicle-associated Membrane Protein-associated Protein B
MCU: Mitochondrial Calcium Uniporter
MiCU: Mitochondrial Calcium Uniporter Family Members
MCUR1: Mitochondrial Calcium Uniporter Regulatory Protein 1
VSMC: Vascular Smooth Muscle Cells
LDL: Low-density Lipoprotein
PCSK9: Proprotein Convertase Subtilisin/Kexin Type 9
SREBP2: Sterol Regulatory Element-binding Protein 2
CAV1: Caveolin-1
ACSL4: Acyl-CoA Synthetase Long-chain Family Member 4
FACL4: Fatty Acid-CoA Ligase 4
PEMT2: Phosphatidylethanolamine N-methyltransferase 2
PE: Phosphatidylethanolamine
PC: Phosphatidylcholine
PINK1: PTEN-induced Kinase 1
EB: Transcription Factor EB
mTOR: Mechanistic Target of Rapamycin
AMPK: AMP-activated Protein Kinase
